# A Quality Assessment of YouTube Videos on Chalazia: Implications for Patient Education and Healthcare Professional Involvement

**DOI:** 10.7759/cureus.78043

**Published:** 2025-01-27

**Authors:** Mübeccel Bulut, Ali Hakim Reyhan

**Affiliations:** 1 Ophthalmology, Necip Fazıl City Hospital, Kahramanmaraş, TUR; 2 Ophthalmology, Harran University, Şanlıurfa, TUR

**Keywords:** chalazion surgery, discern, global quality score (gqs), journal of the american medical association (jama), youtube

## Abstract

Background: YouTube has become a popular source of health information for patients. However, the quality and reliability of videos related to a chalazion, a common eyelid condition, have not yet been thoroughly evaluated.

Research design and methods: One hundred YouTube videos were evaluated using keywords such as "chalazion surgery" and "chalazion removal". Two ophthalmologists assessed the videos using the DISCERN, the Journal of the American Medical Association (JAMA), and Global Quality Scale (GQS) scoring systems and recorded the source of each video.

Results: Analysis revealed low average DISCERN (33.0), JAMA (2.0), and GQS (2.5) scores, indicating poor quality and reliability. Most videos (64%) were classified as "poor" quality, with only a small percentage rated "good" or "excellent". Higher-quality content exhibited positive correlations with engagement metrics.

Conclusion: YouTube videos concerning a chalazion generally lack quality and credibility and are not sufficient for providing reliable patient information. This study emphasizes the need for healthcare professionals to refer patients to trustworthy sources for the creation of high-quality, unbiased content.

## Introduction

A chalazion is a chronic lipogranulomatous inflammatory lesion that occurs in one or several glands in the upper or lower eyelids and is caused by the obstruction of gland orifices and the stagnation of sebaceous secretions. It is one of the most common eyelid diseases and affects individuals of all ages [1]. Approximately 25-50% of chalazia resolve spontaneously or within the first three months after medical treatment [2]. Eyelid hygiene, warm compresses, antibiotic ointment, steroid injection, lesion excision with curettage, and total excision are the recommended therapeutic options [3].

People widely use the internet to obtain medical information [4,5]. According to recent studies, the purpose of 80% of internet users is to get medical information [6].

YouTube is the world’s largest media-sharing website and the second most commonly used site worldwide. It provides free of charge, online video sharing and is the leading information-sharing tool [7,8]. It has approximately one billion users and attracts some six billion views every month according to recent reports. Physicians, healthcare providers, patients, and healthcare institutions all share videos for advertising and educational purposes. However, the quality of the videos may vary and may be misleading [9,10].

Because YouTube is an easily accessible resource; it is used more and more every day to obtain medical information [6]. Although this is a great opportunity for accurate information to be disseminated to large masses, if incorrect and incomplete information is given, it will not benefit people but may be harmful [11-14].

There are studies in the literature that evaluate the quality of YouTube videos about different diseases [8,9-11,13]. However, we could not find any study evaluating the quality of videos about chalazia. We wanted to evaluate the quality of videos about chalazia, the most common disease of the eyelid.

## Materials and methods

Since the videos are shared with everyone on YouTube, we did not need to obtain ethics committee approval. A search was performed using the keywords “chalazion surgery”, “chalazion removal”, “chalazion incision”, “drainage of chalazion", and “chalazion excision” on YouTube on 10 September 2024.

We added 300 frequently watched videos using our search words on YouTube. We excluded videos that were similar, closed to comments and likes-dislikes, and videos under 20 seconds.

The number of views, comments, likes and dislikes, the time since the upload (age), the length of the video (length), the view rate (the number of views per day), and the source of the videos (doctor, patient, health channel, or health care institute) were recorded for each video by a technical assistant (ME). All videos were scored using the DISCERN, Journal of the American Medical Association (JAMA), and Global Quality Score (GQS) systems and misinformation assessment. The videos were evaluated and scored in a double-blind manner by two experienced ophthalmologists (MB and AHR) from different institutions, who collaborated effectively through regular virtual meetings and digital platforms. To minimize potential bias, the ophthalmologists were blinded to the source and context of the videos. The simultaneous assessments were conducted independently, and mean scores were calculated to ensure standardized evaluation metrics.

This study did not require ethical approval as it did not involve human subjects or animal experiments. Although informed consent was not required, all procedures were conducted in accordance with the ethical principles outlined in the Declaration of Helsinki.

Scoring system

DISCERN is a scoring system developed by Oxford University. It consists of three sections and 16 questions (Table 1). In the first part, the reliability of the videos is evaluated by the presence of references given. The second part looks at how many different treatment methods are mentioned. In the third section, the video is evaluated in general. According to the DISCERN scoring system, if the video has a score of 63-75, it is excellent, with 51-62 as good, 39-50 as reasonable, 27-38 as bad, and 15 as very bad [15].

**Table 1:** The DISCERN scoring system

Question Number	What is investigated?	Question Rating				
		No Partially			Yes	
Section 1	Is the publication reliable?					
1	Are the aims clear?	1	2	3	4	5
2	Does it achieve its aims?	1	2	3	4	5
3	Is it relevant?	1	2	3	4	5
4	Is it clear what sources of information were used to compile the publication (other than the author or producer)?	1	2	3	4	5
5	Is it clear when the information used or reported in the publication was produced?	1	2	3	4	5
6	Is it balanced and unbiased?	1	2	3	4	5
7	Does it provide details of additional sources of support and information?	1	2	3	4	5
8	Does it refer to areas of uncertainty?	1	2	3	4	5
Section 2	How good is the quality of information regarding treatment choices?					
9	Does it describe how each treatment works?	1	2	3	4	5
10	Does it describe the benefits of each treatment?	1	2	3	4	5
11	Does it describe the risks of each treatment?	1	2	3	4	5
12	Does it describe what would happen if no treatment is applied?	1	2	3	4	5
13	Does it describe how the treatment choices affect overall quality of life?	1	2	3	4	5
14	Is it clear that there may be more than one possible treatment option?	1	2	3	4	5
15	Does it provide support for shared decision making?	1	2	3	4	5
Section 3	Overall rating of the publication					
16	Based on the answers to all of these questions, rate the overall quality of the publication as a source of information about treatment choices	1, 2, 3 Low Moderate			4, 5 High	

JAMA scores are used to evaluate the quality of information provided by websites. It consists of four questions given 0 or 1 point; 4 indicates high quality (Table 2) [16].

**Table 2:** JAMA score JAMA = Journal of the American Medical Association

JAMA Score	
Authorship	Authors and contributors, their affiliations, and relevant credentials should be provided.
Attribution	References and sources for all content should be listed clearly, and all relevant copyright information should be noted.
Disclosure	Website “ownership” should be prominently and fully disclosed, as should any sponsorship, advertising, underwriting, commercial funding arrangements or support, or potential conflicts of interest.
Currency	Dates, when content was posted and updated, should be indicated.

GQS was developed by Bernard et al. to evaluate its general quality. Videos with a score of 4 or 5 were considered high quality, 3 points were considered medium, and 1 or 2 points were considered low quality (Table 3) [17].

**Table 3:** Global Quality Scoring systems and misinformation assessment

Question Number	Global Quality Score	Misinformation Assessment Score
1)	Poor quality, very unlikely to be of any use to patients	Good explanation of the topic
2)	Poor quality but some information present, of very limited use to patients	Indications are clear
3)	Suboptimal flow, some information covered but important topics missing, somewhat useful to patients	Good execution of the procedure
4)	Good quality and flow, most important topics covered, useful to patients	Compliance with general guidelines
5)	Excellent quality and flow, highly useful to patients	Pathological cases are shown

Misinformation scores using a five-point Likert scale (1 = strongly disagree, 5 = strongly agree) were applied to evaluate five distinct aspects of content quality and accuracy in medical videos. The assessment criteria included the quality of explanation of the subject, clarity of medical indications, procedure execution quality, compliance with general guidelines, and inclusion of pathological cases. The scoring mechanism employed an inverse relationship between the aggregate score and misinformation level, with lower scores indicating higher levels of misinformation, thus permitting a comprehensive evaluation of content reliability [18] (Table 3).

Statistics

Statistical Product and Service Solutions (SPSS, version 22; IBM SPSS Statistics for Windows, Armonk, NY) was used for statistical analysis. Compatibility with normal distribution was evaluated using the Kolmogorov-Smirnov test. Categorical variables were presented as numbers and percentages (%). The Mann-Whitney U test was applied to data meeting non-parametric conditions between two groups. Correlations were determined using Spearman’s correlation test. p values <0.05 were regarded as significant.

## Results

One hundred videos were subjected to evaluation using various keywords on YouTube. The search term "chalazion surgery" was most frequently applied, comprising 48% of queries, followed by "chalazion removal" at 18%, "chalazion incision" at 15%, "chalazion excision" at 12%, "drainage of a chalazion" at 5%, and "chalazion treatment" and "steroid and antibiotic injections" at 1% each.

Table 4 presents the descriptive characteristics of these videos. The median view count was 36,500, ranging from 29 to 2,668,369. Median values for likes and comments were 120.50 (range: 0-8,100) and 1.0 (range: 0-985), respectively. Fifty-nine percent of the videos originated from physicians, 30% from healthcare institutes, and 11% from health channels. The median video length was 2.02 minutes, with a range of 0.30-7.37 minutes. The age of the videos varied widely, with a median value of 600 days and a range of 30 to 14,600 days. This study also assessed various quality metrics. The median DISCERN score was 33.0 (range: 20-63), indicating moderate information quality. The JAMA score had a median value of 2.0 (range: 0-4), suggesting room for improvement in meeting standard criteria for health information. The GQS exhibited a median value of 2.5 (range: 1-4), indicating average overall quality. Finally, the median misinformation score was 10.5 (range: 5-23).

**Table 4:** Descriptive characteristics of the chalazion videos analyzed (N=100)

Variables	Value median (min-max)
View count (n)	36,500 (29-2,668,369)
Likes (n)	120.50 (0-8100)
Comments (n)	1.0 (0-985)
Healthcare institution, n(%)	30 (30%)
Physician, n(%)	59 (59%)
Health channel, n(%)	11 (11%)
Video length (min)	2.02 (0.30-7.37)
Age (days)	600 (30-14,600)
View rate	59.0 (0.5-5640)
DISCERN	33.0 (20-63)
JAMA	2.0 (0-4)
GQS	2.5 (1-4)
Misinformation assessment	10.5 (5-23)

Table 5 shows a comparison of YouTube chalazion video metrics between physician and non-physician content creators. Both groups registered similar view counts, with median values of 36,000 for non-physicians and 38,000 for physicians (p=0.458). Likes and comments were also comparable, with median values of 113.0 likes and 1.0 comments for non-physicians and 103.0 likes and no comments for physicians (p=0.700 and p=0.120, respectively). The only significant difference was in terms of video length, with physician videos being longer (median: 2.37 minutes) than non-physician videos (median: 1.19 minutes, p<0.001). Other metrics, including video ages, view rates, and DISCERN, JAMA, GQS, and misinformation scores, exhibited no significant intergroup differences.

**Table 5:** Comparison of YouTube chalazion video metrics between non-physician and physician content creators The Mann- Whitney U test was used. p<0.05 was considered significant.

Variables	Group 1, non-physician (n=41 videos) Median (min-max)	Group 2, physician (n=59 videos) Median (min-max)	P	U
View count (n)	36,000 (29-2,668,369)	38,000 (104-1,100,000)	0.458	1103.5
Likes (n)	11.0 (2-8100)	103.0 (0-5600)	0.700	1187.0
Comments (n)	1.0 (0-985)	0 (0-309)	0.120	351.0
Video length (mins)	1.19 (0.3-6.34)	2.37 (0.31-7.37)	<0.001	868.0
Age (days)	600 (30-4380)	600 (30-14,600)	0.743	1077.5
View rate	62.0 (0.5-2000)	42.0 (0.5-5640)	0.930	1018.5
DISCERN	32.0 (24-63)	33.0 (20-50)	0.294	1086.0
JAMA	2.0 (0-4)	2.0 (1-3)	0.081	985.5
GQS	2.0 (2-4)	3.0 (1-4)	0.639	1149.0
Misinformation assessment	10 (5-23)	11 (5-19)	0.293	1061.5

Table 6 shows the correlations among DISCERN scores, JAMA scores, GQS, view rates, video age, view counts, and likes. Positive correlations were observed between DISCERN and metrics such as view rate, view counts, likes, and GQS. A significant positive correlation was also determined between GQS and JAMA. GQS also exhibited positive correlations with view rates, view counts, and likes. Video age was positively correlated with both view counts and likes. Additionally, a positive correlation was observed between misinformation scores and DISCERN, GQS, and view rate.

**Table 6:** Correlation matrix of quality metrics (DISCERN, JAMA, and GQS) and engagement metrics (view rate, view count, likes, and age) for YouTube chalazion videos Correlations were determined using Spearman's correlation test.

	DISCERN	JAMA	GQS	View rate	View count	Likes	Age	Misinformation
DISCERN		r=0.235	r=0.510	r=0.332	r=0.269	r=0.212	r=0.074	p=0.527
		p=0.19	p<0.001	p<0.001	p=0.007	p=0.034	p=0.463	p<0.001
JAMA	r=0.235		r=0.337	r=-0.080	r=-0.047	r=0.031	r=-0.013	r=0.125
	p=0.19		p<0.001	p=0.431	p=0.644	p=0.762	p=0.896	p=0.216
GQS	r=0.510	r=0.337		r=0.348	r=0.307	r=0.287	r=0.158	r=0.501
	p<0.001	p<0.001		p<0.001	p=0.002	p=0.004	p<0.001	p<0.001
View rate	r=0.332	r=-0.080	r=0.348		r=0.898	r=0.740	r=0.237	r=0.225
	p<0.001	p=0.431	p<0.001		p<0.001	p<0.001	p=0.17	p=0.024
View count	r=0.269	r=-0.047	r=0.307	r=0.898		r=0.813	r=0.522	r=0.137
	p=0.007	p=0.644	p=0.002	p<0.001		p<0.001	p<0.001	p=0.173
Likes	r=0.212	r=0.031	r=0.287	r=0.740	r=0.813		r=0.400	r=0.089
	p=0.034	p=0.762	p=0.004	p<0.001	p<0.001		p<0.001	p=0.378
Age	r=0.074	r=-0.013	r=0.158	r=0.237	r=0.522	r=0.400		r=0.002
	p=0.463	p=0.896	p=0.116	p=0.17	p<0.001	p<0.001		p=0.985
Misinformation assessment	r=0.527	r=0.125	r=0.501	r=0.225	r=0.137	r=0.089	r=0.002	
	p<0.001	p=0.216	p<0.001	p=0.024	p=0.173	p=0.378	p=0.985	

**Figure 1:**
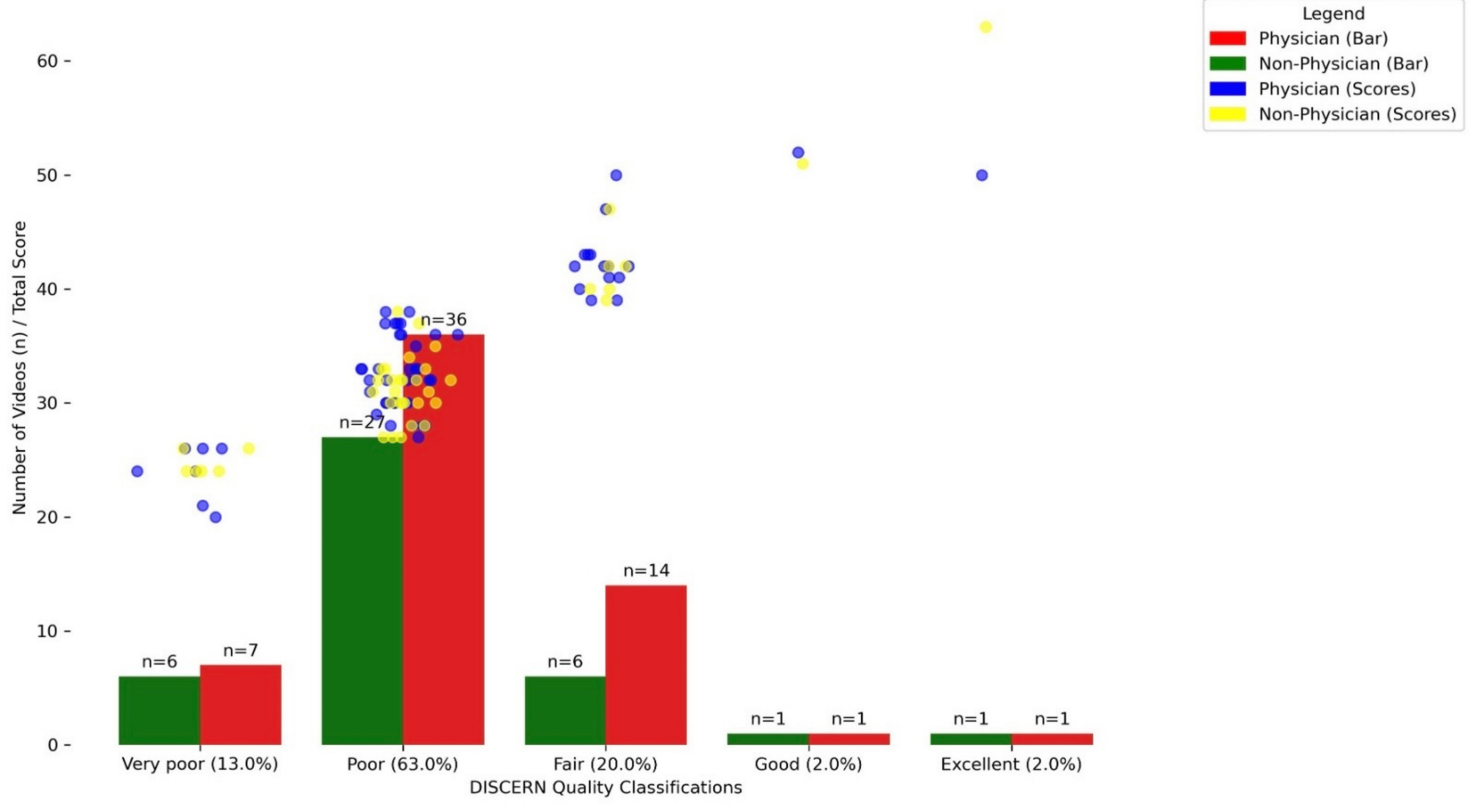
DISCERN quality classifications and total score distribution

As shown in Figure 1, the distribution of chalazion video quality was assessed using the DISCERN criteria, revealing significant differences between physician and non-physician-sourced material. The majority of videos were classified as "poor", accounting for 63% of the total, with marked contributions from both physician and non-physician sources. "Fair" quality videos comprised 20% of the total, and "very poor" videos comprised 13%. Only a small fraction of the videos were rated "good" (2%) or "excellent" (2%). The scatter plot further illustrates the variability in DISCERN scores within each classification, highlighting the preponderance of lower-quality content.

## Discussion

This study critically examined the quality and reliability of YouTube videos on the subject of chalazia, highlighting the platform's role as a significant, yet often, unreliable source of health information. To the best of our knowledge, no previous investigators have examined the quality of YouTube material concerning chalazia. Since we searched for videos related to surgical methods in chalazion treatment, we did not include videos other than those uploaded by doctors, health channels, and health institutions. Fifty-nine of the 100 videos analyzed were uploaded by physicians and 41 by health institutions or channels. The descriptive statistics of the 100 videos are shown in Table 5. The average video values were 33.0 for DISCERN, 2.0 for JAMA, and 2.5 for GQS. We used these scores to classify the quality of YouTube videos. These scores suggest that chalazion surgery videos on YouTube are generally of low quality. Similarly, Yildiz et al. showed that soft contact lens videos on YouTube were of poor quality and reliability [19]. Videos on the subjects of retinal detachment surgery, multifocal intraocular lenses, and pterygium surgery have also been described as being of low quality [20,21]. Our analysis reveals that the reliability and quality of YouTube videos on the subject of chalazia are low, consistent with previous studies that highlight a lack of comprehensive and accurate information [19-22]. This is particularly concerning in light of YouTube's popularity as a source of health information, on which videos by non-medical professionals frequently present biased or incomplete information, potentially misleading patients. While YouTube's accessibility makes it a valuable tool for patient education, the absence of standardized quality control can lead to misinformation, unrealistic expectations, or inappropriate self-diagnosis and treatment.

Additionally, one of the primary factors contributing to the low scores in our analysis of YouTube chalazion videos was the evaluation criteria focusing on the inadequate provision of comprehensive information regarding the procedure and its potential adverse effects, insufficient elaboration of treatment choices, and limited documentation of whether the interventions were performed under appropriate clinical conditions.

"Chalazion surgery" was the most frequently used search term in this study, representing 48% of queries. This finding echoes the observations of Erdem et al. [8] in their study of thulium laser enucleation of the prostate (ThuLEP) videos, in which the authors noted that many YouTube videos were more focused on surgical technique rather than patient education. This suggests a broader trend in medical YouTube content, where videos may not always align with patients’ information needs [22].

The most important indicator of the popularity of a YouTube video is the view count [23,24]. Interestingly, our chalazion video analysis revealed no significant differences in view counts, likes, or comments between physician and non-physician videos.

The analysis of YouTube videos from the perspective of medical content, specifically comparing physician and non-physician creators, has been a subject of particular interest in recent studies. Our analysis of chalazion videos revealed no statistically significant difference in quality measures such as DISCERN, JAMA, and GQS scores between physician and non-physician content creators. This appears to indicate that both groups struggled to provide high-quality, reliable information on chalazia. Similarly to our own findings, a study of pterygium videos by Ozturkmen et al. observed no significant difference in quality measures such as DISCERN, JAMA, and GQS scores between physician and non-physician content creators [25]. This finding contrasts with a fibromyalgia study, in which physicians were found to produce content that was mostly of high and medium quality (70.2%), while non-medical users largely produced low-quality content (86.7%) [26]. This discrepancy suggests that the quality gap between physician and non-physician creators may vary depending on the medical topic or condition being discussed.

The longer durations of videos uploaded by physicians may be attributable to their wish to provide more information without seeking to advertise. No difference was found between physicians and other uploaders in terms of other parameters. Healthcare professionals play a pivotal role in enhancing the quality of online medical information, particularly on platforms such as YouTube. As Altunel et al. suggest, medical experts should actively create and curate high-quality, unbiased video content that addresses patient concerns in a comprehensive manner [20]. Ramadhani et al. emphasized the importance of physicians recognizing the limitations of online videos and directing patients toward more reliable sources of information. This dual approach involving content creation and patient guidance is crucial to ensuring access to trustworthy information, ultimately supporting informed decision-making in healthcare [27].

The analysis of DISCERN quality classifications for YouTube videos on a chalazion reveals a concerning trend in the quality of health information available online. The distribution of videos across quality categories revealed a significant skew toward lower-quality content, with the majority of videos falling into the "poor" category (Figure 1). Similarly to our findings, Sledzińska et al. reported that the overall quality of meningioma treatment videos was poor, with only 16.4% being rated as "good" or "excellent” [28]. This trend is particularly pronounced among physician-uploaded videos, which counterintuitively dominate the lower-quality categories. Conversely, videos classified as "good" or "excellent" are markedly scarce, indicating a substantial gap in high-quality, reliable health information on this platform. This prevalence of poor-quality videos, especially those from ostensibly authoritative sources such as physicians, raises serious concerns about the reliability of online health resources. This situation may lead to misinformation, potentially affecting patient understanding and decision-making on the subject of chalazion treatment. The scarcity of high-quality content also suggests a critical need for improvement in the creation and curation of health-related videos. By focusing on creating more "good" and "excellent" quality videos, content creators can improve viewer engagement and also contribute to a more reliable and trustworthy online health information ecosystem.

Correlation analysis of the YouTube chalazion videos reveals a complex interplay between quality and engagement metrics. DISCERN and GQS scores exhibited moderate-to-strong positive correlations with one another and with engagement metrics such as view rate and view count, suggesting that higher-quality content tends to attract more viewers and engagement. Interestingly, these quality metrics also correlated negatively with misinformation, indicating their potential role in identifying reliable health information. JAMA scores, however, exhibited a weaker correlation with engagement metrics, implying that these may not align as closely with viewer preferences. Engagement metrics (view rate, view count, and likes) were strongly correlated with one another, reinforcing the interconnected nature of viewer interaction and approval. The age of the video exhibited weaker correlations overall, highlighting that content quality and relevance, rather than longevity, represent key drivers of popularity on YouTube. These findings underscore the importance of producing high-quality, reliable content in order to effectively engage viewers and combat misinformation in online health videos.

The study has several limitations. First, the analysis was limited to videos in English, and the inclusion of material in other languages might have provided a more comprehensive understanding of the global landscape of chalazion-related content on YouTube. Second, the study did not evaluate the actual impact of these videos on patient knowledge, attitudes, or behaviors, which may constitute an important area for future research. Furthermore, the lack of detailed analysis regarding the specific content issues contributing to the low quality of videos limits the applicability of the findings in addressing these deficiencies effectively.

## Conclusions

Our analysis of YouTube videos on the subject of chalazia reveals a concerning preponderance of low-quality and unreliable content, with the majority of videos, regardless of their source, failing to meet adequate standards of medical information dissemination. This study highlights the need for healthcare professionals not only to guide patients toward more credible information sources but also to actively contribute to the creation and curation of high-quality, unbiased video content on platforms such as YouTube. The research also highlights the critical need for healthcare professionals to enhance the quality of YouTube content on the subject of chalazia, since improved online medical information can significantly bolster patient education and informed decision-making, ultimately leading to better health outcomes.
